# Reverse-engineering of gene networks for regulating early blood development from single-cell measurements

**DOI:** 10.1186/s12920-017-0312-z

**Published:** 2017-12-28

**Authors:** Jiangyong Wei, Xiaohua Hu, Xiufen Zou, Tianhai Tian

**Affiliations:** 1 School of Statistics and Mathematics, Zhongnan University of Economics and Law, Wuhan, 430073 China; 2 School of Computer, Central China Normal University, Wuhan, 430079 China; 3 School of Mathematics and Statistics, Wuhan University, Wuhan, 430072 China; 4 School of Mathematical Sciences, Monash University, Melbourne, VIC 3800 Australia

**Keywords:** Genetic regulatory network, Blood stem cell, Single-cell experiment, Graphic model, Dynamic model

## Abstract

**Background:**

Recent advances in omics technologies have raised great opportunities to study large-scale regulatory networks inside the cell. In addition, single-cell experiments have measured the gene and protein activities in a large number of cells under the same experimental conditions. However, a significant challenge in computational biology and bioinformatics is how to derive quantitative information from the single-cell observations and how to develop sophisticated mathematical models to describe the dynamic properties of regulatory networks using the derived quantitative information.

**Methods:**

This work designs an integrated approach to reverse-engineer gene networks for regulating early blood development based on singel-cell experimental observations. The wanderlust algorithm is initially used to develop the pseudo-trajectory for the activities of a number of genes. Since the gene expression data in the developed pseudo-trajectory show large fluctuations, we then use Gaussian process regression methods to smooth the gene express data in order to obtain pseudo-trajectories with much less fluctuations. The proposed integrated framework consists of both bioinformatics algorithms to reconstruct the regulatory network and mathematical models using differential equations to describe the dynamics of gene expression.

**Results:**

The developed approach is applied to study the network regulating early blood cell development. A graphic model is constructed for a regulatory network with forty genes and a dynamic model using differential equations is developed for a network of nine genes. Numerical results suggests that the proposed model is able to match experimental data very well. We also examine the networks with more regulatory relations and numerical results show that more regulations may exist. We test the possibility of auto-regulation but numerical simulations do not support the positive auto-regulation. In addition, robustness is used as an importantly additional criterion to select candidate networks.

**Conclusion:**

The research results in this work shows that the developed approach is an efficient and effective method to reverse-engineer gene networks using single-cell experimental observations.

**Electronic supplementary material:**

The online version of this article (doi:10.1186/s12920-017-0312-z) contains supplementary material, which is available to authorized users.

## Background

The advances in omics technologies have generated huge amount of information regarding gene expression levels and protein kinase activities. The availability of the large datasets provides unprecidental opportunities to study large-scale regulatory networks inside the cell by using various types of omics datasets [1, 2]. The majority of the generated datasets are based on the measurements using a population of cells. However, biological experiments and theoretical studies have suggested that noise is a very import factor to determine the dynamics of biological systems [3–5]. In recent years, a number of experimental tools including single-cell qPCR, single-cell RNA sequencing, and multiplex single-cell proteomic methods, have been used for lineage tracing of cellular phenotypes, understanding cellular functionality, and high-throughput drug screening [6–8]. A centre theme in single-cell study is the highly heterogeneity at virtually all molecular levels beyond the genome [9]. The availability of large amount single-cell data has stimulated great interests in bioinformatics studies for analysing, understanding and visualizing single-cell data. A particular interesting research problem is the development of regulatory network models using single-cell observation data [10–12].

Mathematical methods for the analysis of single-cell observation data is mainly for normalization of experimental data, identification of variable genes, sub-population identification, differentiation detection and pseudo-temporal ordering [13]. These top-down approaches are mainly based on statistical analysis and machine learning techniques, and thus are able to deal with large-scale single-cell datasets [14]. For example, the algorithm Wanderlust represents each cell as a node and then ensemble cells into k-nearest neighbour graphs [15]. For each graph, it computes iteratively the shortest-path distance between cells. Another important type of approaches is the graphic model that represents the connection and/or regulations between genes and proteins. Different methods, such as the probabilistic graphic model, linear regression model, Bayesian network and Boolean network, have been applied to develop the regulatory networks [16–21].

One of the major challenges in computational biology is the development of dynamic models, such as differential equation models, to study the dynamic properties of genetic regulatory networks [17, 22, 23]. There are two major steps in designing a dynamic model, namely to determine the structure of network by specifying the connection and regulation between genes and proteins [19], and to quantify the strength of regulations [24]. In the last decade, a number of approaches have been applied to design dynamic models, including differential equation model, neural network model, petri-network model, and chemical reaction systems [25–29]. Recently we have proposed an integrated framework that combines both the probabilistic graphic model and differential equation model to infer the p53 gene networks that regulating the apoptosis process [30]. Regarding single-cell data, an additional step is to develop the pseudo-temporal trajectory by assuming each cell is uniformly distributed at different time points of the evolutionary process. A number of methods have been developed to analyse single-cell data [15, 31–35]. For example, The dimensionality reduction methods and cellular trajectory learning technique have been used to reverse-engineer the regulatory network by using differential equation based models [31]. In addition, the Single Cell Network Synthesis(SCNS) toolkit has been designed to develop regulatory network using single-cell experimental observation [36, 37]. Although these methods use either logistic models or dynamic models to infer genetic networks, a number of issues still remains, such as inference of network structure, development of appropriate dynamic models, and estimation of model parameters in the dynamic model.

To address these issues, this work propose a novel approach to reverse-engineer gene networks using single-cell observations. To get pseudo-temporal ordering of single cells, we first use a method of dimensionality reduction, namely diffusion maps [36, 37], to get the lower dimensional structure of gene expression data, and then use the wanderlust algorithm [15] to order single cells according to their relative position in the cell cycle. Since there is substantial noise in the generated pseudo-trajectory data, the Gaussian processes regression method is used to smooth the expression data [38]. Then the GENIE3 algorithm is employed to infer the structure of gene regulatory network [39]. Using single-cell quantitative real-time reverse transcription-PCR analysis of 33 transcription factors and additional marker genes in 3934 cells with blood-forming potential, we develop a graphic model for the network of 40 genes and dynamic model for a network of nine genes.

## Methods

### Experimental data

A recent experimental study used the single-cell qPCR technique to identify the expression levels of 46 genes in 3934 single stem cells that were isolated from the mouse embryo [37]. The Flkl expression in combination with a Runx1-ires-GFP report mouse was used to measure cells with blood potential at distinct anatomical stages across a time course of mouse development. Single Flkl ^+^ cells were flow sorted at primitive streak (PS), neural plate (NP) and head fold (HF) stages. In addition, the E8.25 cells were subdivided into putative blood and endothelial populations by isolating GFP ^+^ cells (four somite, 4SG) and Flkl ^+^ GFP ^−^ cells (4SFG ^−^), respectively. Cells were sorted from multiple embryos at each time point, with 3934 cells going to subsequent analysis. Experimental study quantified the expression of 33 transcriptional factors involved in endothelial and hematopoietic development, nine marker genes, including the embryonic globin *Hbb-bH1* and cell surface marker such as *Cdh5* and *Itga2b* (CD41), as well as four reference housekeeping genes (i.e. *Eif2b1, Mrpl19, Polr2a and UBC*). We select 40 genes from this dataset but exclude four house-keeper genes and two other genes (i.e. *HoxB2* and *HoxD8*) because the variations in the expression levels of these fix genes are relative small.

The dCt values in Supplementary Table 3 [37] represent the relative gene expression levels. These values will be employed as experimental data to reverse-engineer the regulatory network of the remaining 40 genes in this work. Note that the majority of these dCt values are negative. It would be difficult to use a dynamic model to describe the data with negative values. Therefore we conduct a shift computation by assuming that the minimal dCt value is zero in “Mathematical modelling” subsection.

### Pseudo-temporal ordering

The process of pseudo-temporal ordering can be divided two major steps. The first step uses Diffusion Maps for lower-dimensional visualization of high-dimensional gene expression data, then the Wanderlust algorithm is employed to order the individual cells to get the trends of different genes.

Diffusion Map is a manifold learning technique for dealing with dimensionality reduction by re-organizing data according to their underlying geometry. It is a nonlinear approach of visual exploration and describes the relationship between individual points using lower dimension structure that encapsulates the data [31]. An isotropic diffusion Gaussian kernel is defined as

1 $$ W_{\varepsilon}(x_{i},x_{j})=\exp\left(-\frac{\|x_{i}-x_{j}\|^{2}}{2\varepsilon}\right),  $$

where $x_{i}=\left (x_{i}^{(1)},\ldots,x_{i}^{(D)}\right), i=1,\ldots,N$, is the expression data of gene *i*, *D* is the number of single-cell, ∥·∥ is the Euclidean norm, and *ε* is an appropriately chosen kernel bandwidth parameter which determines the neighbourhood size of points. In addition, an *N*×*N* Markov matrix normalizing the rows of the kernel matrix is constructed as follows:

2 $$ M_{ij}=\frac{W_{\varepsilon}\left(x_{i},x_{j}\right)} {P(x_{i})},  $$

where *P*(*x*_*i*_) is an normalization constant given by $P(x_{i})=\sum _{j} W_{\varepsilon }(x_{i},x_{j})$. *M*_*ij*_ represents the connectivity between two data points *x*_*i*_ and *x*_*j*_. It is also a measure of similarity between data points within a certain neighbourhood. Finally we compute eigenvalues and eigenvectors of this Markov matrix, and choose the largest *d* eigenvalues. The corresponding *d* eigenvectors are the output as the lower dimensional dataset *Y*_*i*_,*i*=1,…*d*.

Using the generated low dimensional dataset, we use the Wanderlust algorithm to get a one-dimensional developmental trajectory. There are several assumptions about the application Wanderlust to sort gene expression data from single cells. Firstly, the data sample includes cells of entire developmental process. In addition, the developmental trajectory is linear and non-branching, it means that the developmental processes is only one-dimensional. Furthermore, the changes of gene expression values is gradual during the developmental process, and thus the transitions between different stages are gradual. Based on these assumptions, we can infer the ordering of single cells and identify different stages in the cell development by using the Wanderlust algorithm.

The Wanderlust algorithm also consists of two major stages, namely an initiation step and an iterative step for trajectory detection. In the first stage, we select a set of cells as landmarks uniformly at random. Each cell will have landmarks nearby. Then we construct a *k*-nearest-neighbours graph that every cell connect to *k* cells that are most similar to it, then we randomly pick *l* neighbours out of the *k*-nearest-neighbours for each cell and generate a *l*-out-of- *k*-nearest-neighbours graph (l-k-NNG). Then the second stage begins for the trajectory detection. One early cell point *s* should be selected first in this algorithm, which serves as the starting point of psuedo-trajectory. The point *s* can be determined by the Diffusion Maps method in the first. For every single cell, the initial trajectory score can be calculated as the distance from the starting-point cell *s* to that cell.

For each cell *t* with early cell *s* and landmark cell *l*, if *d*(*s,t*)<*d*(*s,l*), then *t* precedes *l*, otherwise *t* follows *l* in the pseudo-trajectory. For each landmark cell, we define a weight as

3 $$ w_{l,t}=\frac{d(l,t)^{2}}{\sum_{m} d(l,m)^{2}},  $$

and the trajectory score for *t* is defined as

4 $$ {Score}_{t} = \sum_{l}\frac{d(l,t)}{n_{l}}w_{l,t},  $$

where *n*_*l*_ is the number of landmark cells and the summation is over all landmarks *l*. The scores also include the beginning cell and landmarks. Then we use trajectory score as a new orientation trajectory and repeat the orientation step until landmark positions converge.

### Data smooth

The pseudo-trajectory gene expression data determined by the Wanderlust algorithm have a large variations in the gene expression levels. Thus we use the Gaussian processes regression method for smoothing the noisy data. The Gaussian processes regression is a generative non-parametric approach for modelling probability distributions over functions. It begins with a prior distribution and updates this distribution when data points are observed, and finally produces the posterior distribution over functions [38].

Assume that we have the ordered data ${\mathcal {D}} =\{\mathbf {t,y}\}$. The observation **y** can be regarded as samples of random variables with the underlying distribution function *f*(**t**), which is described by a Gaussian noise model:

$$\mathbf{y}= f(\mathbf{t})+\boldsymbol{\epsilon},\boldsymbol{\epsilon}\sim N\left(\mathbf{0},\sigma^{2} {I}\right). $$

We want to make prediction of the system state *y* ^∗^ at a point *t* ^∗^ based on the above model. The joint distribution of **y** and *y* ^∗^ is:

$$\left[ \begin{array}{c} \mathbf{y}\\ y^{*} \end{array}\right] \sim N\left(0, \left[\begin{array}{cc} K & K_{*}^{T}\\ K_{*} & K_{**} \end{array}\right] \right) $$

Here *K* is a kernel trick to connect two observations. One popular choice for the kernel is the squared exponential covariance function, defined by

$$K\left(t,t^{\prime}\right)=\sigma^{2} \exp\left[\frac{-\left(t-t^{\prime}\right)^{2}}{2l^{2}}\right] $$

where *σ* ^2^ is a signal variance parameter and *l* is a length scale parameter. If *t*≈*t* ^′^, it means that *f*(*t*) is highly correlated with *f*(*t* ^′^). However, if *t* is distant from *t* ^′^, *K*(*t,t* ^′^)≈0, the two points is largely uncorrelated with each other. Finally the posterior distribution is given by *y* ^∗^|**y**∼*N*(***μ***,***Σ***) with:

$$ \begin{aligned} \boldsymbol{\mu} &= K_{*}\left[K+\sigma^{2} I\right]^{-1}y\\ \boldsymbol{\Sigma} &= K_{**}-K_{*}\left[K+\sigma^{2} I\right]^{-1}K_{*}^{T} \end{aligned}  $$

### Networks construction

In this work the GENIE3 algorithm will be employed for reconstruction of regulatory network using the determined pseudo-temporal trajectory based on single-cell data in [40]. Instead of considering the regulation in a whole network, this method studies the inference accuracy of *N* genes separately by using the regression model. In this regression model, the expression level of a particular gene is described by the regulatory function that is determined by the expression of the other *N*−1 genes, given by

5 $$ x_{t+1}^{(j)}=f_{j}\left(\boldsymbol{x_{t}}^{(-j)}\right)+\epsilon,  $$

where **x** ^(−*j*)^=(*x* ^(1)^,…,*x* ^(*j*−1)^,*x* ^(*j*+1)^,…,*x* ^(*N*)^)^*T*^, *ε* is a random noise with zero mean, and function *f*_*j*_ is designed to search for genes that regulate the expression of gene *x* ^(*j*)^ using a random forest method. The function only exploits the expression in **x** ^−*j*^ of the genes that are direct regulators of gene *j*, i.e. genes that are directly connected to gene *j* in the targeted network. For each gene, a learning sample is generated with the expression levels of that gene as the output by using and expression levels of all other genes as input. A function is learned from the data and a local ranking of all genes except *j* is computed. The *N* local rankings are then aggregated to get a global ranking of all regulatory links.

The nature of function *f*_*j*_ is unknown but they are expected to involve the expression of several genes (combinatorial regulation) and to be non-linear. Each subproblem, defined by a learning sample, is a supervised (non-parametric) regression problem. Using square error loss, each problem amounts at finding a function that minimizes the following error:

6 $$ \sum_{k=1}^{N} \left(x_{k}^{j}-f_{j}\left(x_{k}^{-j}\right)\right)^{2}.  $$

Note that, depending of the interpretation of the weight, the aggregation to get a global ranking of regulatory links is not trivial. It requires to normalize each expression vector appropriately in the context of this tree-based method.

### Mathematical modelling

We have designed a modelling framework by using differential equations to study the genetic regulations based on microarray gene expression data [30]. This general approach is be extended to develop dynamic models using single-cell expression data. Using the notations introduced in previous subsection, the expression levels of the *i*-th gene is denoted as *x*_*i*_(*t*) at time *t*. The evolution of gene expression levels is described by the following dynamic model using differential equations, given by

7 $${} \frac{d x_{i}}{dt}=c_{i}+k_{i}f\left(x_{1},\ldots,x_{N}\right)-d_{i} x_{i}, \qquad i=1,\ldots,N  $$

where *c*_*i*_ and *k*_*i*_ are the basal and maximal synthesis rate of gene *i* in gene expression, respectively, *d*_*i*_ is the decay rate of transcript. The key point is the selection of the regulatory function, which should include both positive and negative regulations appropriately. Based on the Shea-Ackers’ formula [41], this work uses the following function

8 $$ f_{i}=\frac{a_{i1}x_{1}(t)+\ldots+a_{iN}x_{N}(t)}{1+b_{i1}x_{1}(t)+\ldots+b_{iN}x_{N}(t)}  $$

Coefficients *a*_*ij*_ represents regulation from gene *j* to the expression of gene *i*. This regulation may be positive (*a*_*ij*_>0) or negative (*a*_*ij*_=0) if the corresponding coefficient (*b*_*ij*_>0). For example, if *a*_*ii*_>0, a larger value in the expression level of gene *j* will promote the expression level of gene *i*. However, if *a*_*ij*_=0, a higher expression level of gene *j* will only increase the value of denominator of the regulatory function and thus decrease its value. This model assumes that, if *b*_*ij*_=0, then the coefficient *a*_*ij*_ must be zero. In addition, it may be no regulatory relationship from gene *j* to gene *i* if (*a*_*ij*_=*b*_*ij*_=0). Since there is no time scale for experiment, is is assumed that each cell in the pseudo-trajectory is a unit time. Thus the time period of model is [ 0,3933] since there are 3934 single cells.

Note that the proposed model (7) is based on the assumption that the expression levels *x*_*i*_(*t*)≥0. However, the majority of the experimental data are negative. To address this issue, we conduct a linear transformation in order to change the negative values of dCt to positive values, denoted as *dCt*_1_. For each gene, we assume the minimal value of the dCt values is zero and the shift computation is

$$dCt_{1} (gene\; i)= dCt_{0}(gene\;i) +|min(dCt_{0}(gene\;i))| $$

It is clear that the transformed value *dCt*_1_ is always non-negative.

We use the Approximate Bayesian Computation (ABC) rejection sampling algorithm [42, 43] to search for the optimal model parameters. The uniform distribution is used as the prior distribution of the unknown parameters. Since the value of *dCt*_1_ may be quite large, we use the relative absolute error the measure the difference between simulations and experimental data, given by

9 $$ E=\sum_{i=1}^{N}\sum_{j=1}^{M} \frac{|x_{ij}-x_{ij}^{*}|}{\max_{j}{\left\{x_{ij}^{*}\right\}}},  $$

where $x_{ij}^{*}$ and *x*_*ij*_ are the simulated and experimentally measured gene expression levels at time point *t*_*j*_ for gene *i*, respectively. Due to the large number of single cells, which leads to a long range of time period for numerical simulation of model (7), the proposed model is stiff and simulation frequently breaks down when the search space is not very small. Thus instead of obtaining simulation of the whole time interval, we consider the numerical solution over *k* pseudo-time points and calculate the transfer probability

$$\begin{aligned} L(\theta)=f_{0}\left[(t_{0}, x_{0})|\theta\right]\prod_{i=1}^{N/k} f\left(\left[\left(t_{ki},x_{ki}\right)|\left(t_{k(i-1)+1},x_{k(i-1)+1}\right); \theta\right]\right), \end{aligned} $$

In this work we choose *k*=100 and assume that *f*_0_[(*t*_0_,*x*_0_)|*θ*]=1 and the transitional probability is calculated by using the absolute error kernel, given by

$$f\left(\left[\left(t_{ki},x_{ki}\right)|\left(t_{k(i-1)+1},x_{k(i-1)+1}\right); \theta\right]\right)=\frac{1}{E_{i}} $$

where *E*_*i*_ is the simulation error (9) using (*t*_*k*(*i*−1)+1_, *x*_*k*(*i*−1)+1_) as the initial condition.

Since different sets of estimated model parameters may generate simulations with similar simulation error, we use the robustness property of the mathematical model as an additional criterion to select the estimated model parameters [44, 45]. The detailed computing process of robustness analysis can be found in [30]. For each module of gene regulation, we use the ABC algorithm to generate a number of sets of model parameters, and then select the top 5 sets that have the minimal estimation error for robustness analysis. In this way, we are able to exclude the influence of simulation error on the robustness property of the model.

## Results and discussion

### Diffusion map visualization

**Fig. 1:**
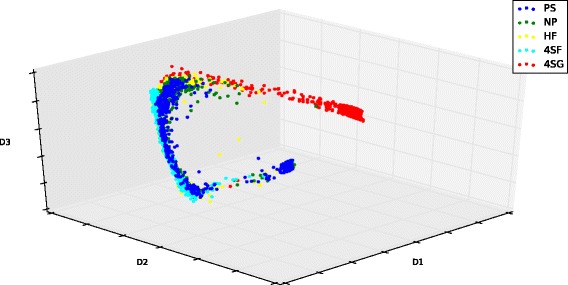
Diffusion map of 3934 single cells using the qPCR data

Using the single cell data, a Diffusion Map algorithm is first employed to visualize the dataset [31]. The purpose of this step is to reduce the dimension of dataset and provide the pattern of the data in the three-dimensional space. Generally we choose three eigenvectors of the kernel matrix (2) for visualization. These three eigenvectors are those that have the largest eigenvalues. Figure 1 gives the diffusion coordinates for the first, second and third largest eigenvalues. It shows that all the data points in the development trajectory form only one branch. This result shows that the single cell dataset is appropriate for generating a pseudo-temporal trajectory by using the Wanderlust algorithm. This analysis also provides a single cell that will be used the first cell in the development of the pseudo-temporal trajectory.

### Wanderlust ordering

**Fig. 2:**
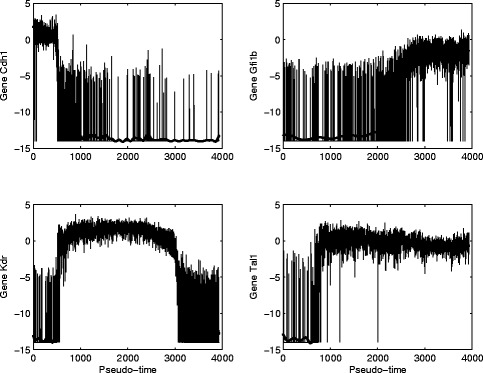
Pseudo-expression trajectory of four genes using raw qPCR data and smoothed data (solid-bold line)

After determining the starting cell *s* in the previous section, the Wanderlust algorithm is then employed to obtain the pseudo-temporal trajectory for the expression dynamics of genes. In this program, the widely used Euclidean measure is used to calculate the distance between different cells. Figure 2 provides the determined pseudo-temporal trajectory of four genes based on the raw dCt data in [31]. Based on the generated pseudo-temporal trajectory, we find that the expression levels of the four housekeeping genes are barely changed. Similar observations can also be applied to the expression levels of genes *HoxB2* and *HoxD8*. Therefore we will not consider these six genes in the network development.

The pseudo-temporal trajectory has large variations in the expression levels of every gene. Thus it is very difficult to use differential equation model to realize such data with large variation. To address issue, the Gauss process regression method is used to remove the variations in the data and produce more smooth trajectory. Figure 2 also provides the pseudo-temporal trajectory after the smooth process for the four genes. Compared with the raw dCt data with large variations, the smoothed data for the same gene have much smaller fluctuations in the expression levels.

One characteristics of the expression levels is that all genes (excluding the six genes) have both high and low expression levels. Genetic switching exists in the expression levels. Based on the time interval with high or low expression levels, the processed data can be classified into a number of patterns. For example, gene *Cdh1* has high expression levels in the pseudo-time interval [ 0,500] and low expression levels in the following time interval. However, genes *Gfi1b* and *Tal1* have low expression level in the pseudo-time interval [ 0,2500] and [ 0,800], respectively, but the expression level is switched to high levels in the following time intervals. A few of genes, such as gene *Kdr* in Fig. 2, have two genetic switchings in the pseudo-temporal trajectory. Since our proposed technique has been used to realize the similar observations for genetic switching [45], this modelling approach will be used in this work to realized the pseudo-temporal trajectories.

### Networks construction

**Fig. 3:**
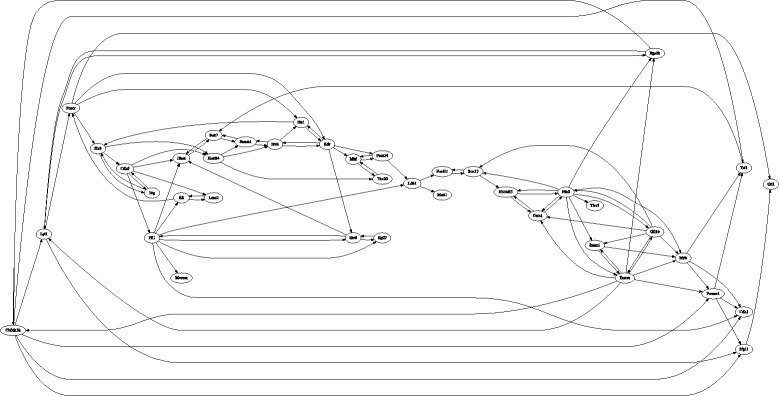
Regulatory structure of network with 40 genes that is generated by the GENIE3 algorithm

**Fig. 4:**
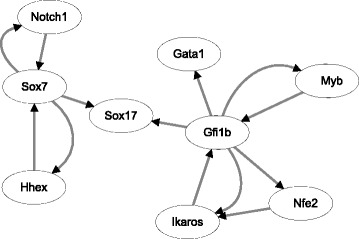
Graphic model for the regulatory network with nine genes. All the regulations (except that for gene pairs Sox7 and Sox17) are derived from the network in Fig. 3

With the availability of pseudo-temporal trajectory based on single-cell data, we then reverse-engineer the network structure for the regulatory network of 40 genes. The GENIE3 algorithm is used to develop a graphic model of these 40 genes. For each gene, a linear regression model is used to infer the regulation of other 39 genes to the expression of that gene. Then we can obtain 39 weight values for the possible regulation strength for each gene and the total number of genetic regulation strength is 1560 (=39×40). In order to maintain a certain number of regulations to each gene, we select the top 10 weight values for each gene. In this way the number of selected regulation values is only 400. Since a network with 400 undirected edges is still very dense, we set up a threshold value to select the top regulation strength. An edge is selected if its weight is larger than the threshold value. We have tested different values of the threshold value by decrease the threshold value gradually. The optimal threshold value is determined if the number of remaining edges is relative small but all genes should be connected to the network. The constructed network is given in Fig. 3. In this network there are 100 regulatory connections between these 40 genes. Among these 40 genes, the maximal number of connection edges for a gene is 11; while the minimal number of edge for a gene is 1. The average number of edge per gene is 2.5 and on average each gene connects to five other genes,

We have developed a mathematical model to describe the dynamics of the network with 40 genes. However, numerical results suggest that it is difficult to use a differential equation model to simulate the expression dynamics of 40 genes. The mathematical model includes a large number of model parameters that should be estimated. Due to the complexity of searching space, the simulation error is large. In addition, the genetic switching in the observation data makes the designed ODE model is stiff. Therefore we consider a small network with a less number of genes. We compare the designed graphic model in Fig. 3 and the model in Figure 3C in [37], and then select nine genes, namely *GATA1, Gfi1b, Hhex, Ikaros, Myb, Nfe2, Notch1, Sox7* and *Sox17*. The regulation relationship between these nine genes in these two networks are consistent. However, the regulation between these nine genes in our graphic model in Fig. 3 is not a fully connected network, thus the regulation between the gene pair (it Sox7, Sox17), which exists in the network in [37], was added to Fig. 4 in order to form a complete network. The developed network model is presented in Fig. 4.

### Mathematical model

The nine genes selected in Fig. 4 is divided into two groups based on their expression patterns. In these nine genes, there are five genes, namely *GATA1, Gfi1b, Ikaros, Myb, Nfe2*, whose expression are activated at the pseudo-time point *t*=∼2300 and their expression activities are promoted a high level with different speeds for different genes. However, the expression of the remaining four genes, namely *Hhex, Notch1, Sox7 and Sox17*, is first inhibited and their expression levels go down to a low level at he pseudo-time point *t*=∼700; but their expression is activated at *t*=∼2500 and the expressions return to the high levels again. The observed gene expression changes are consistent with other experimental observations showing that genes *GATA1, Gfi1b* and *Ikaros* do have substantial changes of the expression levels over time [45]. The genetic switching in the expression levels of these genes is important to maintain the functional activities of blood stem cells. Using the proposed modelling method in [45], it is assumed that some key model parameters are variables of time rather than a constant. For the five genes in the first group, we use the following synthesis rate for gene expression, given by

$$k_{i}=\left\{ \begin{array}{ll} k_{i0}, & t<2500\\ k_{i0}*A_{i} & else \end{array}\right. $$

where *k*_*i*0_ is the basal synthesis rate of gene *i*, and *A*_*i*_>1. Regarding the four genes in the second group, it is assumed that both synthesis rate and degradation rate are variables of time, since we need to realize the first switching from high expression level to low expression level,

$$k_{i}=\left\{ \begin{array}{ll} k_{i0}*A_{i}, & 500<t<1000\\ k_{i0} & else \end{array}\right. $$

$$d_{i}=\left\{ \begin{array}{ll} d_{i0}*A_{i}, & 2500<t<3000\\ d_{i0} & else \end{array}\right. $$

**Fig. 5:**
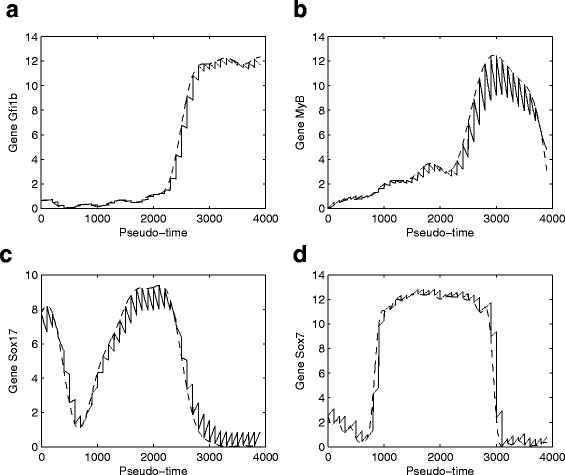
Expression levels of four genes based on the network in Fig. 4 (solid-line: the experimental data obtained by Gaussian process regression; dash-line: expression levels predicted by mathematical model)

Our simulation results suggest that the proposed ODE system is still even when an implicit method with very good stability property is used for numerical solution of the proposed model. Numerical simulation will break down if we try to find the solution over a relatively long pseudo-time interval. Therefore we have to separate the whole time interval into a number of subintervals, and in each subinterval, we use the experimental observation data as the initial condition to generate solution of the subinterval. Figure 5 shows simulation results using a fixed time period of 100 unit time. We have also examined other lengths of time period, namely *t*=50 and *t*=200. Numerical results are consistent with those showing Fig. 5. In addition, the ABC algorithm is used to infer the unknown model parameters using the data shown in Fig. 2. Figure 5 suggests that the proposed model is able to match experimental data very well. Certainly the simulation error is dependent on the length of subinterval. The simulation error is larger if the length of subinterval is larger.

### Inference of network with more regulations

The proposed network in Fig. 4 includes nine one-way or mutual regulations. It is fully consistent with the network predicted in [37]. To make predictions about the potential regulations among these nine genes, we extend the network by including more regulations. We apply the GENIE3 algorithm to the raw dCt data of these nine genes only. According to the calculated weight of the target edges, we select the highest weight of 27 one-way regulations. Since there are a few two-way regulations in the selected 27 regulation edges, the generated network includes 17 un-directional regulations (see Additional file 1). Compared with the network in Fig. 4, the number of potential regulation edges has been doubled. The structure of the extended network is shown in Additional file 1: Figure S1 in Supplementary Information. Note that the regulations in Additional file 1: Figure S1 do not include all the regulations in Fig. 4. The added regulation (*Sox 7, Sox 17*) in Fig. 4 does not appear in Additional file 1: Figure S1. However, the other eight regulations in Fig. 4 also appears in Additional file 1: Figure S1.

**Fig. 6:**
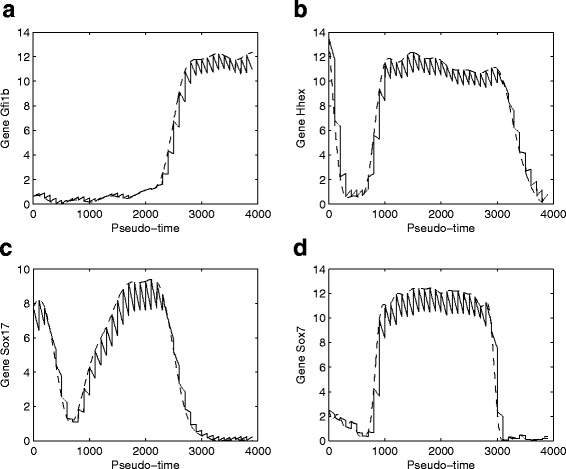
Expression levels of four genes based on the extended network in Additional file 1: Figure S1 (solid-line: the experimental data obtained by Gaussian process regression; dash-line: expression levels predicted by mathematical model)

**Fig. 7:**
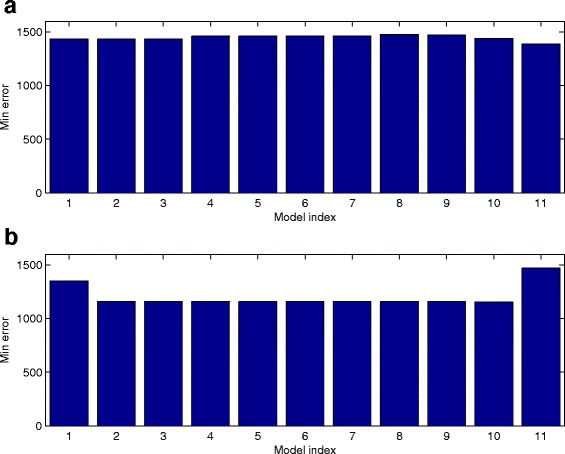
Accuracy of the inferred network models without and with positive auto-regulations. **a** The accuracy of the inferred network models based on the network in Fig. 6. **b** The accuracy of the inferred network models based on the network in Additional file 1: Figure S1. (model index: 1. Network without aotu-regulation, 2 ∼ 10. Network with auto-positive regulation for genes *Gata1, Gfi1b, Hhex, Ikaros, Myb, Nfe2, Notch1, Sox17, Sox7*, respectively, 11. All nine genes have positive positive auto-regulation

For this extended network, we also use the ABC algorithm to infer model parameters using the modified dCT values. Simulation results of four genes are presented in the Fig. 6. Numerical results for the total simulation error suggest that the extended network (see Fig. 7 b, index 1) has better accuracy than the network in Fig. 4 (Fig. 7 a, index 1). Note that the model based on a network with more regulations has more model parameters, which gives more flexibility to match the experimental data. Thus it is reasonable that the model based on network in Additional file 1: Figure S1 has better accuracy than that in Fig. 4. However, this simulation result suggests that, compared with the network in Fig. 4, more regulations may exist.

### Inference of network with auto-regulation

In the graphic model generated by the GENIE3 algorithm, the auto-regulation, namely the positive or negative regulation of a gene to the expression of itself, is not considered. To find the potential auto-regulations in these genes, we test the network by adding positive auto-regulation to a particular gene. For the *i*-th gene, we set *b*_*ii*_>0; and the value of *a*_*ii*_ is *a*_*ii*_>0 for positive auto-regulation. We first test the gene network shown in Fig. 4 with positive auto-regulation for only one gene. Numerical results in Fig. 7 a suggest that no module with positive auto-regulation (model index 2 ∼ 10) has smaller simulation error than that of the network without any auto-regulation (index 1 in Fig. 7 a). We have also tested the network in which each gene has positive auto-regulation. Results in (index 11 in Fig. 7 a) shows that simulation error of this module is larger than that of any other module in Fig. 7 a. Thus it is unlikely that all these genes have positive auto-regulation.

We have also conducted the positive auto-regulation test for the model based on the network in Additional file 1: Figure S1. An interesting result is that, compared with the network without auto-regulation (index 1 in Fig. 7 b), the model with positive auto-regulation to any one gene (model index 2 ∼ 10) has better accuracy than the network model without auto-regulation. Note that, compared with the network model without auto-regulation, the network model with positive auto-regulation to one gene has only two additional model parameters. The small change in the number of unknown parameters would not bring much flexibility to match experimental data. Thus this result suggests that there may be positive auto-regulation for some genes in this network. However, when we add auto-regulation to all genes (index 11 in Fig. 7 b), similar to the result in (index 11 in Fig. 7 a), the simulation error of this module is larger than any other module. This gives further evidence that it is unlikely that all these genes have positive auto-regulation.

### Robustness analysis

**Fig. 8:**
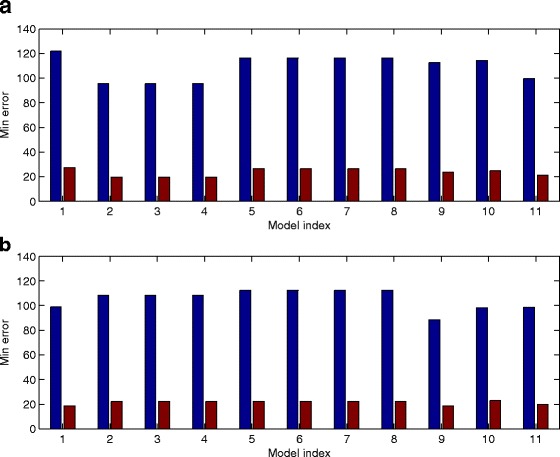
Robustness property of various models for the gene network with nine genes. **a** Robustness for the gene network in Fig. 4. **b** Robustness for the gene network with 17 regulations (Model index: 1: netwok without any auto-regulation, 2:10: positive auth-regulation for gene *Gata1, Gfi1b, Hhex, Ikaros, Myb, Nfe2, Notch1, Sox17, Sox7*, respectively, 11: all nine genes have positive auto-regulation). For each model, the first bar is the mean of error; while the second bar is the variance of error

We also test the robustness property of the developed models and the models with positive auto-regulations. We first use the estimated optimal parameter set to generate one simulation which is regarded as the exact simulation of the model without any perturbation. Then all the model parameters are perturbed by using a uniformly distributed random variable, and perturbed simulations are obtained using the perturbed model parameters. We generate 1000 sets of perturbed simulations and calculate the mean and variance of the simulation error for the perturbed simulation over the unperturbed simulations. For the gene network shown in Figs. 4 and 8 a suggests that the models with auto-regulation for genes *Gata1, Gfi1b, Hhex* have better robustness property than the model without any perturbation. However, for the gene network with 17 regulations in Additional file 1: Figure S1, the networks with auto-regulation for genes *Sox7, Sox17* have better robustness property than the network without positive auto-regulation. These simulation results do not provide strong evidence to support the positive auto-regulation in the nine genes in Fig. 4.

## Conclusion

In this work we have designed an integrated approach to reverse-engineer gene networks for regulating early blood development based on singel-cell experimental observations. The diffusion map method is firstly used to obtain the visualization of gene expression data derived from 3934 stem blood cells. The wanderlust algorithm is then employed to develop the pseudo-trajectory for the activities of a number of genes. Since the gene expression levels in the developed pseudo-trajectory show large fluctuations, we then use Gaussian process regression method to smooth the gene express data in order to obtain pseudo-trajectory with much less fluctuations. The proposed integrated framework consist of both the GENIE3 algorithm to reconstruct the regulatory network and a mathematical model using differential equations to describe the dynamics of gene expression. The developed approach is applied to study the network regulating early blood cell development, and we designed a graphic model for a regulatory network with forty genes and a differential equations model for a network of nine genes. The research results in this work shows that the developed approach is an efficient and effective method to reverse-engineer gene networks using single-cell experimental observations.

In this work we use simulation error as the key criterion to select the model parameters and infer the regulation between genes. However, because of the complex searching space of model parameters and noise in experimental data, it may be difficult to judge which model is really better than others if the difference between simulation errors is small. In fact, simulation errors of various models for the network of nine genes are quite close to each other. Therefore, in addition to using simulation error as the unique criterion to select a model, other measurements, such as AIC value, parameter identifiability and robustness property of a network, are also needed as important criteria. All of these issues are potential topics for future research.

## Additional file

Additional file 1
**Figure S1.** Extended gene network with 17 regulations. (DOCX 76 kb)
