# Supplementary material for: Reverse-engineering of gene networks for regulating early blood development from single-cell measurements
Source: BMC Med Genomics. 2017 Dec 28;10(Suppl 5):72. doi: 10.1186/s12920-017-0312-z (PMC5751697; doi:10.1186/s12920-017-0312-z)
Supplement: Additional file 1 — Figure S1. Extended gene network with 17 regulations. (DOCX 76 kb) [file 12920_2017_312_MOESM1_ESM.docx]

**Supplementary Information**

**Reverse-engineering of gene networks for regulating early blood development from single-cell measurements**

**Jiangyong Wei, Xiaohua Hu, Xiufen Zou and Tianhai Tian**

**
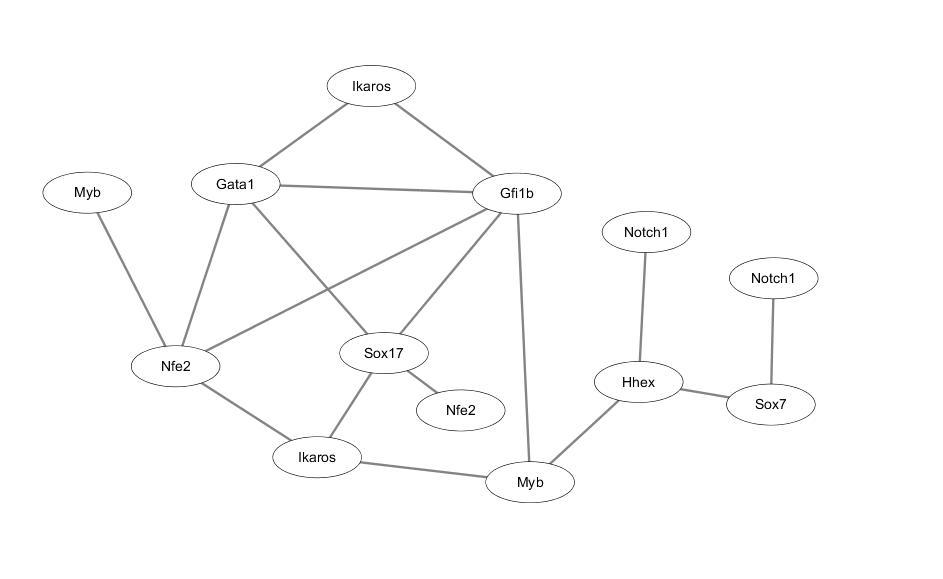
**

**Figure S1.** Extended gene network with 17 regulations. This network includes the network in Figure 4 in the paper but does not include the added regulation between gene pairs (Sox7, Sox17) in Figure 4.
